# Variant-informed multimodal tissue-state modeling reveals an NRF2 stress niche and dependency axis in KEAP1/NFE2L2-altered cancer

**DOI:** 10.3389/fimmu.2026.1957511

**Published:** 2026-10-06

**Authors:** Mowei Kong, Qiuing Li, Xingwei Zhao, Yu Wang, Yamei Luo, Yang Yu, Chunxiang Zhang

**Affiliations:** 1 Department of Cardiology, The Affiliated Hospital of Southwest Medical University, Luzhou, Sichuan, China; 2 Key Laboratory of Medical Electrophysiology, Ministry of Education, Institute of Cardiovascular Research, Luzhou, Sichuan, China; 3 The Key Laboratory of Optimal Utilization of Natural Medicine Resources (The Union Key Laboratory of Guiyang City-Guizhou Medical University), School of Pharmaceutical Sciences, Guizhou Medical University, Guiyang, Guizhou, China; 4 School of Medical Information and Engineering, Southwest Medical University, Luzhou, China; 5 Basic Medicine Research Innovation Center for Cardiometabolic Diseases, Ministry of Education, Southwest Medical University, Luzhou, Sichuan, China; 6 Nucleic Acid Medicine of Luzhou Key Laboratory, Luzhou, Sichuan, China

**Keywords:** cancer immunotherapy, KEAP1/NFE2L2 alterations, multimodal tissue-state modeling, NRF2 stress response, tumor microenvironment and immune contexture

## Abstract

**Introduction:**

Cancer genotypes influence transcriptional programs, spatial organization, histopathology, and treatment-response states, yet these molecular and tissue-level features are often analyzed separately in public-data studies. VISTA was developed as a variant-informed, multimodal framework for modeling genotype-linked tissue states and prioritizing validation-ready biological hypotheses.

**Methods:**

VISTA integrates somatic variants, protein-region and AlphaMissense-informed priors, RNA-state scoring, whole-slide image embeddings, immunotherapy cohorts, spatial and single-cell references, cell-line dependency maps, and pharmacogenomic screens. We evaluated the framework in KEAP1/NFE2L2-altered lung cancer and melanoma, using the NRF2 stress axis as a biologically defined test case. Analyses incorporated project-adjusted modeling, bootstrap confidence intervals, permutation testing, leave-project sensitivity analyses, independent tumor-level validation cohorts, fixed-effect external meta-analysis, model-system analyses using DepMap/CCLE, tissue-context analyses, immunotherapy cohorts, and exploratory whole-slide image analyses.

**Results:**

In TCGA LUAD, LUSC, and SKCM pilot cohorts, KEAP1 and NFE2L2 alterations were consistently associated with NRF2 stress activation across project-adjusted models and sensitivity analyses. Independent analyses of CPTAC LUAD, CPTAC LSCC, and non-TCGA LUAD cohorts reproduced higher NRF2-state scores in KEAP1/NFE2L2-altered tumors. Fixed-effect external meta-analysis supported associations with both KEAP1 mutation and combined KEAP1-or-NFE2L2 alteration status. DepMap/CCLE analyses provided complementary model-system evidence: KEAP1/NFE2L2-mutant models had higher NRF2 scores, while NRF2-high or KEAP1/NFE2L2-mutant contexts showed stronger NFE2L2 dependency. Tissue-context analyses positioned NRF2 stress near hypoxic, stromal, myeloid, and immune programs. Immunotherapy cohorts supported the relevance of NRF2-associated states to treatment-response biology, while also indicating limited utility for static pan-cancer prediction. Exploratory whole-slide image analyses using pathology foundation-model embeddings identified morphology associations with myeloid, hypoxic, and CAF/TGF-beta barrier programs.

**Discussion:**

VISTA provides an evidence-layered approach to variant-informed tissue-state modeling. By integrating genomic, transcriptomic, imaging, spatial, cellular, dependency, and pharmacogenomic evidence, it can help prioritize genotype-linked cancer states and validation-ready hypotheses. VISTA is not intended to function as a stand-alone clinical predictor; its claims are bounded by the evidence available in each data layer and by the biological and clinical context of the analyzed cohorts.

## Introduction

Somatic cancer variants are now catalogued at considerable depth. Variant lists alone, however, rarely explain tissue-scale phenotypes such as stress adaptation, microenvironment remodeling, pathology morphology, immunotherapy response context, or therapeutic vulnerability (1–7). A mutation may perturb a protein; the tumor phenotype arises later, through transcriptional programs, spatial organization, immune and stromal compartments, histologic architecture, and context-specific dependencies (8–11). Computational oncology therefore needs methods that connect variant interpretation to tissue-scale mechanism while keeping the strength of each evidence layer explicit (2–4, 12).

Many public-data studies still treat each modality as a separate analysis (2–4). Mutation-focused studies identify recurrent drivers or predicted variant effects (5, 6, 13, 14). Transcriptomic studies score immune or stress programs (8, 15). Immune-deconvolution resources add complementary TME references (9, 16, 17), pathology studies extract whole-slide image features (10, 18), and weakly supervised or clinical-grade WSI studies provide related precedents (19–21). Single-cell and spatial transcriptomics analyses localize programs to compartments and tissue regions (22–27). Immunotherapy cohorts test response associations, while dependency screens nominate vulnerabilities (1, 12, 28, 29). These layers are informative. When analyzed separately, however, they provide a limited basis for building mechanism chains and can encourage overinterpretation of single-modality associations as causal or clinically actionable (7, 9, 11, 12, 28, 30).

VISTA is a variant-informed multimodal framework that links somatic alterations to matched transcriptional states and integrates evidence from tumor cohorts, tissue context, histopathology, model-system dependencies, and pharmacogenomics. Its tiered, auditable structure supports coherent interpretation of multimodal data and prioritization of mechanisms and therapeutic hypotheses. The current analysis integrates TCGA somatic variation, RNA-seq, whole-slide image manifests, external immunotherapy cohorts, spatial and single-cell data, DepMap/Chronos dependencies, and PRISM/GDSC/CTRP pharmacogenomic screens (1–4, 10–12, 30–38). VISTA does not build patient-specific simulators or prospectively predict treatment response. Its role is to organize public evidence so that variant-linked tissue states, mechanistic hypotheses, and dependency or pharmacogenomic axes can be assessed reproducibly.

We selected the KEAP1/NFE2L2–NRF2 axis to demonstrate VISTA because it provides a well-defined genotype-to-state relationship with relevance across molecular, tissue, and therapeutic evidence layers. KEAP1 and NFE2L2 regulate NRF2 stress biology (39–41), which is linked to oxidative stress adaptation, metabolic rewiring, therapy resistance, and immune/TME remodeling (42–45).modeling The axis therefore provides a demanding test case. A useful framework should recover the known regulatory relationship, quantify its transcriptional effect, place it in tissue-state context, examine response-state relevance and transfer limits, and nominate perturbational vulnerabilities without exceeding what the data can support (12, 45–47).

Here, VISTA identifies a consistent KEAP1/NFE2L2-to-NRF2 stress mechanism across TCGA lung cancer and melanoma cohorts (2–4, 39). The analysis links this mechanism to hypoxic, stromal, myeloid, and immune tissue-state contexts; external immunotherapy response-state analyses; exploratory pathology foundation-model embeddings; DepMap/Chronos NRF2-circuit dependency nominations; and PRISM/GDSC/CTRP drug-response associations (1, 9, 10, 31). Claims are interpreted according to their evidence tier, distinguishing tumor-level replication, model-system support, tissue-context evidence, and exploratory analyses.

## Results

### VISTA constructs a reproducible variant-informed multimodal tissue-state modeling framework

VISTA connects variant interpretation to tissue-scale tumor ecosystem modeling through a sequential multimodal workflow. The workflow links somatic variants and protein-domain or AlphaMissense-supported priors to RNA-state scoring, whole-slide image morphology, immunotherapy response context, spatial and single-cell tissue context, and DepMap/Chronos dependency nomination (**Figure 1a**) (5–9, 13–17, 48). The public input resources, derived compact tables, and manuscript-supporting outputs are summarized in **Figure 1b**. These evidence layers are organized into primary mutation-to-RNA associations, tissue-context support, exploratory morphology associations, model-system dependency support, and pharmacogenomic nominations (**Figure 1c**; **Table 1**). The framework separates discovery, external tumor replication, model-system evidence, perturbation closure, and clinical-inference boundaries (**Figure 1d**).

**Figure 1:**
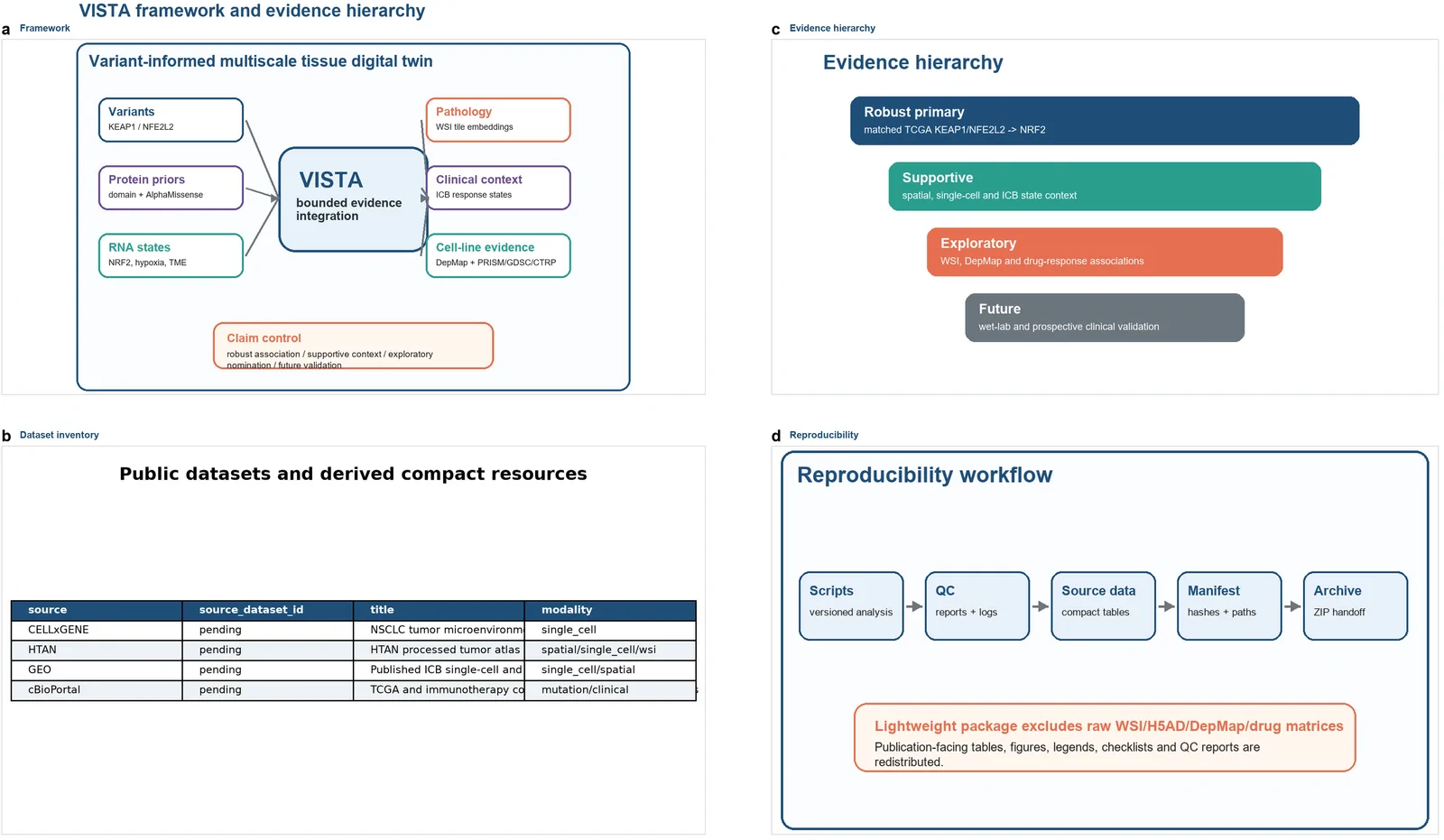
VISTA framework and evidence hierarchy. **(a)** Overview of the VISTA workflow linking somatic variants, protein-domain and AlphaMissense priors, RNA-state scoring, whole-slide image morphology, immunotherapy response context, spatial and single-cell tissue context, and DepMap/Chronos dependency nomination. **(b)** Dataset inventory summarizing public input resources, derived compact tables, and manuscript-supporting outputs used in the computational framework. **(c)** Evidence hierarchy separating robust primary mutation-to-RNA associations, tissue-context support, exploratory morphology associations, model-system dependency support, pharmacogenomic nominations, and validation boundaries. **(d)** Validation-boundary map separating discovery, external tumor replication, model-system evidence perturbation closure, and clinical-inference layers, with the claim scope assigned to each layer.

**Table 1:** VISTA evidence hierarchy.

Evidence layer	Main role in VISTA	Claim boundary
Matched TCGA variant-to-RNA analysis	Primary mechanism-level association	Robust observational association, not causal perturbation
Spatial and single-cell scoring	Tissue-context localization	Supportive context, not genotype-matched causal validation
Immunotherapy cohorts	Response-state relevance and transfer limits	Not a universal static pan-cancer predictor
Prov-GigaPath WSI tile encoder	Morphology-to-molecular association baseline	Tile-encoder pooled baseline, not full LongNet slide inference
DepMap/Chronos	Dependency nomination	Cell-line dependency hypothesis, not therapeutic validation
PRISM/GDSC/CTRP	Drug-response association axes	Pharmacogenomic association evidence, not clinical drug validation

Within this hierarchy, matched TCGA mutation-to-RNA associations provide the primary mechanism-level evidence (2–4). Spatial and single-cell analyses localize the associated transcriptional programs across tissue compartments (22–27), immunotherapy cohorts evaluate response-state relevance (1, 28, 29), and WSI analysis establishes a pathology foundation-model embedding baseline (10, 18–21,62–64). DepMap/Chronos and pharmacogenomic resources extend the framework to model-system dependencies and drug-response associations (11, 12, 30–38, 49–61,65). The role and interpretation of each evidence layer are summarized in **Table 1**.

### Protein-informed variant mapping nominates KEAP1/NFE2L2 as a coherent NRF2 regulatory axis

VISTA first profiled variants across priority cancer genes in the pilot cohorts (**Figure 2a**) and then mapped KEAP1 and NFE2L2 alterations to protein-domain and AlphaMissense-supported contexts (**Figures 2b, c**) (13, 14, 48). KEAP1 variants mapped to regulatory regions involved in NRF2 suppression, whereas NFE2L2 variants mapped to NRF2 regulatory contexts (46, 47). Integrated domain and variant-effect prioritization identified KEAP1/NFE2L2 as the most coherent mechanism axis among the evaluated genes (**Figure 2d**). This prioritization defined the mechanism chain linking KEAP1/NFE2L2 alterations to activation of the NRF2 stress transcriptional program (**Figure 2e**) (39–41, 43–45).

**Figure 2:**
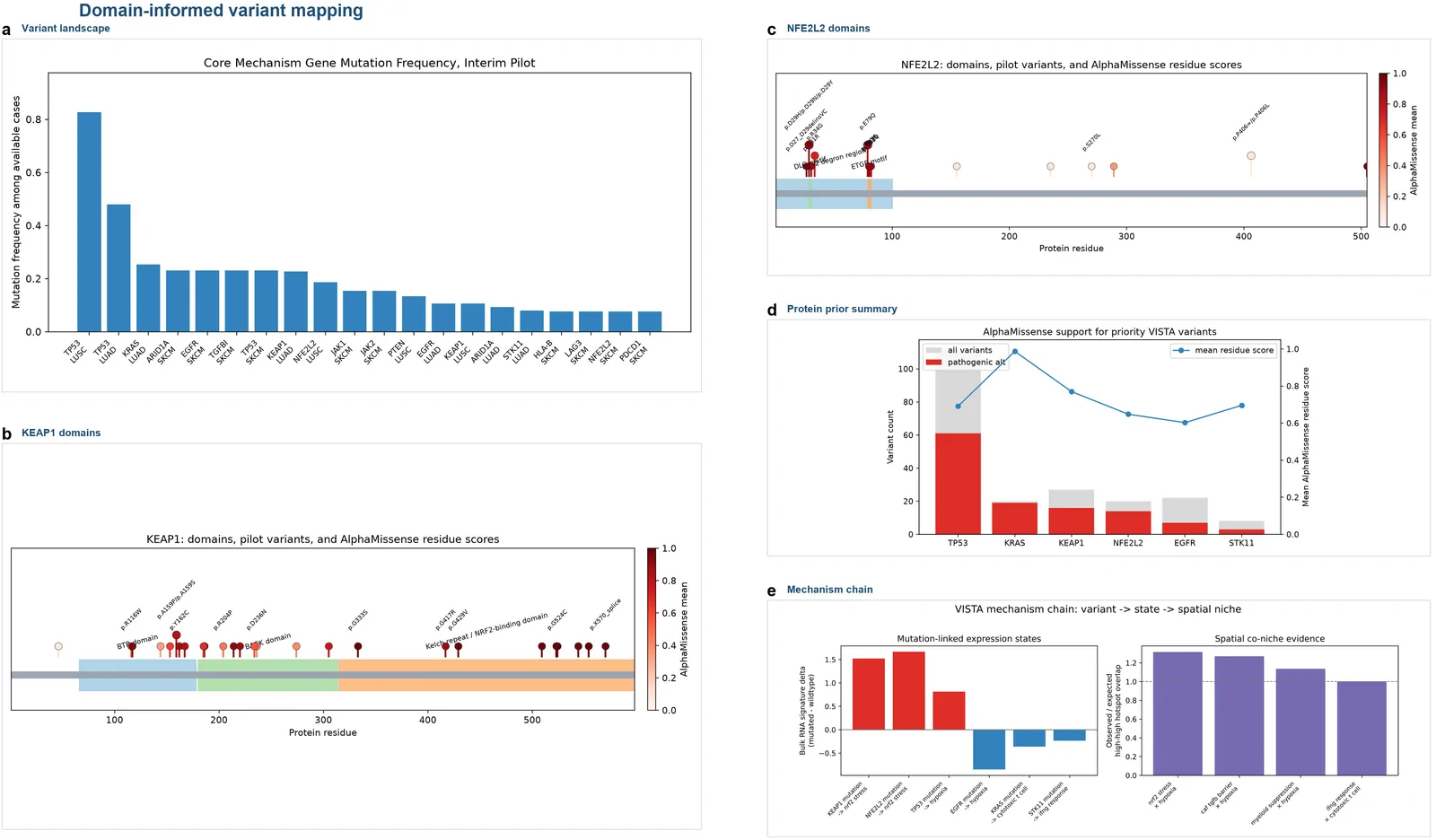
Domain-informed variant mapping nominates KEAP1/NFE2L2. **(a)** Variant profile across priority cancer genes in the pilot cohorts. **(b)** KEAP1 variants mapped to protein-domain and AlphaMissense-prior context. **(c)** NFE2L2 variants mapped to regulatory/domain-prior context. **(d)** Summary of AlphaMissense and domain-informed prioritization across the core mechanism gene set. **(e)** Mechanism-chain schematic linking KEAP1 and NFE2L2 alterations to NRF2 stress programs. These panels support a protein-informed prioritization of the KEAP1/NFE2L2-NRF2 regulatory axis, while preserving the boundary that individual variants are nominated by computational priors and cohort associations rather than exhaustively functionally validated.

This mechanism-first strategy also reduced the risk of presenting VISTA as an overly broad platform (7). VISTA did not test every gene-state combination with equal emphasis. It used protein context to nominate an interpretable axis and then examined whether matched transcriptomic, tissue, response, and dependency layers supported the same mechanism chain (8, 10, 13, 31). The remaining analyses therefore focus on KEAP1/NFE2L2-to-NRF2 as the clearest biological thread in the current dataset (39–41, 45).

### KEAP1/NFE2L2 alterations robustly activate NRF2 stress across TCGA pilots

The central VISTA result was the association between KEAP1/NFE2L2 alteration and NRF2 stress activation. The priority variant-to-state analysis showed increased NRF2 stress in KEAP1-mutant tumors (delta 1.517; Welch FDR 1.38e-05) and NFE2L2-mutant tumors (delta 1.665; Welch FDR 0.00213) (**Figure 3a**) (39–41). Project-stratified score distributions showed the same association across the TCGA-LUAD, TCGA-LUSC, and TCGA-SKCM cohorts (**Figure 3b**) (2–4). Project-adjusted estimates further supported the KEAP1 and NFE2L2 associations, with FDR values of 5.25e-09 and 0.000376, respectively (**Figure 3c**).

**Figure 3:**
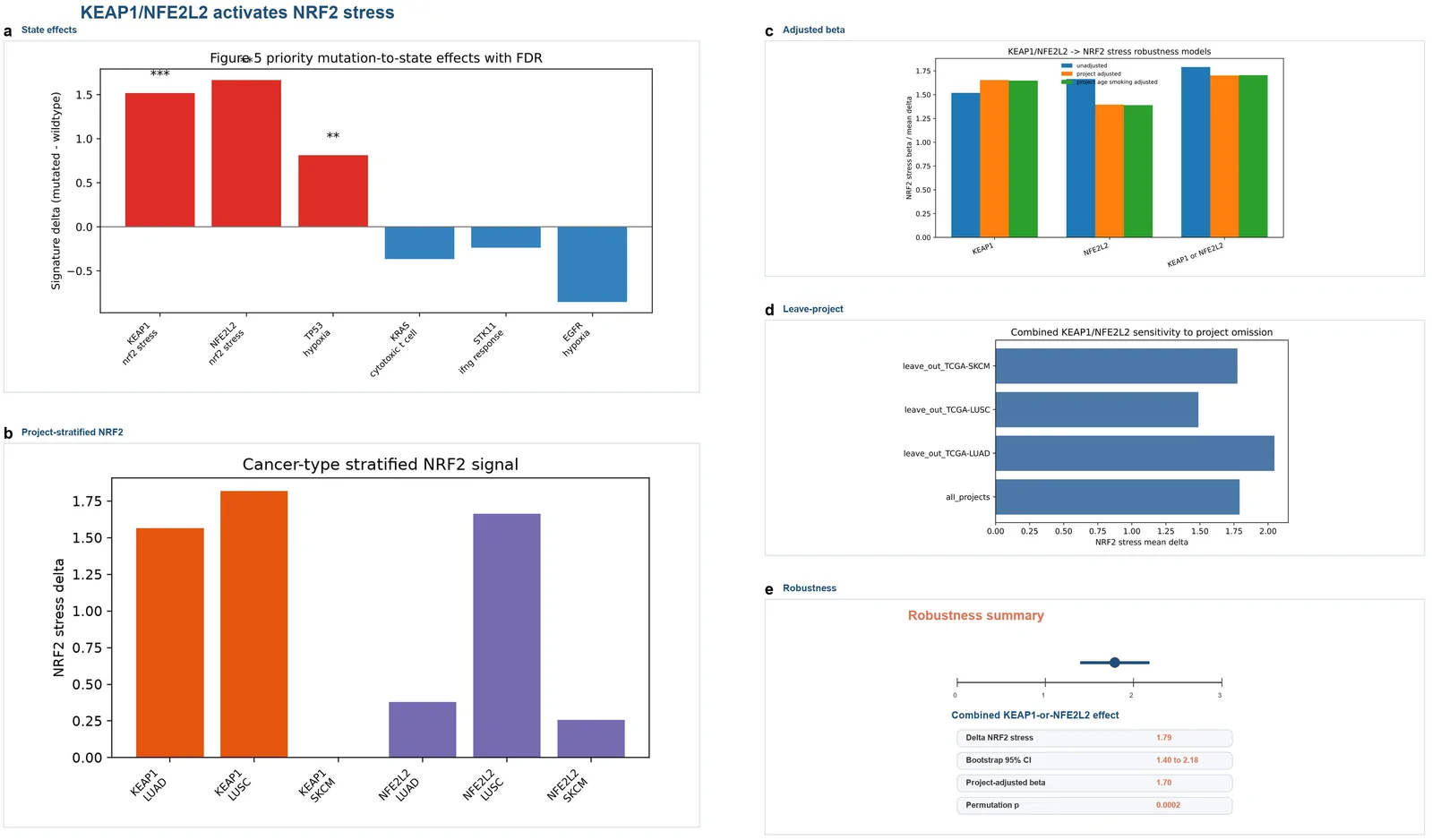
KEAP1/NFE2L2 alterations robustly activate NRF2 stress. **(a)** Priority variant-to-state associations with NRF2 stress shown among KEAP1/NFE2L2-altered tumors. **(b)** Project-stratified NRF2 stress scores by KEAP1/NFE2L2 alteration status. **(c)** Project-adjusted effect estimates for the central mutation-to-state association. **(d)** Leave-project sensitivity analysis evaluating whether the NRF2 association is driven by a single project. **(e)** Bootstrap, model-adjusted and permutation summary for the combined KEAP1-or-NFE2L2 exposure. The combined exposure shows a large NRF2 stress difference, bootstrap confidence interval stability, project-adjusted significance and a permutation result consistent with a low empirical false-positive probability. **P<0.01; ***P<0.001.

Leave-project analysis preserved the direction of the NRF2 association when individual cancer projects were omitted (**Figure 3d**). Across 163 RNA-profiled cases, KEAP1-mutant tumors showed an NRF2-stress difference of 1.52 (bootstrap 95% CI, 1.06–1.96; project-adjusted beta=1.65; p=4.86e-11), and NFE2L2-mutant tumors showed a difference of 1.67 (95% CI, 0.99–2.27; beta=1.40; p=6.96e-06). The combined KEAP1-or-NFE2L2 group showed the largest difference, at 1.79 (95% CI, 1.35–2.17; beta=1.60; p=3.52e-14), with concordant bootstrap, project-adjusted, and permutation results (**Figure 3e**). Together, the five panels identify KEAP1/NFE2L2-to-NRF2 activation as the leading variant-to-state association in the TCGA pilot cohorts.

### Independent tumor cohorts replicate the KEAP1/NFE2L2-to-NRF2 state association

External tumor replication was evaluated in cBioPortal-accessible cohorts with matched mutation and RNA-expression data. Three independent cohorts met the prespecified replication criterion. In CPTAC LUAD, KEAP1-mutant tumors had higher NRF2 scores than wild-type tumors (n=12 versus 98; delta=1.60; bootstrap 95% CI, 1.24 to 1.93; FDR = 5.95e-06). CPTAC LSCC showed higher NRF2 scores for KEAP1 mutation (n=12 versus 68; delta=0.75; 95% CI, 0.43 to 1.08; FDR = 6.82e-04), NFE2L2 mutation (n=9 versus 71; delta=1.04; 95% CI, 0.57 to 1.47; FDR = 4.08e-03), and the combined KEAP1-or-NFE2L2 alteration (n=21 versus 59; delta=1.03; 95% CI, 0.73 to 1.33; FDR = 1.88e-07). OncoSG LUAD similarly showed higher NRF2 scores for KEAP1 mutation (n=6 versus 163; delta=1.87; 95% CI, 1.22 to 2.46; FDR = 3.38e-03) and the combined alteration (n=7 versus 162; delta=1.94; 95% CI, 1.38 to 2.42; FDR = 8.18e-04). LUAD-CAS and SMC lung each included fewer than three altered samples in the relevant contrasts and contributed directionally concordant descriptive evidence. Cohort-level results and their evidence classifications are summarized in **Table 2** and **Supplementary Figure S1**.

**Table 2:** Completed external validation and model-system support results.

Validation layer	Cohort/resource	Contrast	n altered/n WT	Delta NRF2 score (95% CI)	FDR	Evidence grade/boundary
Independent tumor replication	OncoSG LUAD	KEAP1 vs WT	6/163	1.87 (1.22 to 2.46)	3.38e-03	Independent tumor replication
Independent tumor replication	CPTAC LUAD	KEAP1 vs WT	12/98	1.60 (1.24 to 1.93)	5.95e-06	Statistical replication; tumor-level external validation
Independent tumor replication	CPTAC LSCC	KEAP1 vs WT	12/68	0.75 (0.43 to 1.08)	6.82e-04	Statistical replication; tumor-level external validation
Independent tumor replication	CPTAC LSCC	NFE2L2 vs WT	9/71	1.04 (0.57 to 1.47)	4.08e-03	Statistical replication; NFE2L2-specific support
Independent tumor replication	CPTAC LSCC	KEAP1-or-NFE2L2 vs WT	21/59	1.03 (0.73 to 1.33)	1.88e-07	Statistical replication; combined alteration support
Independent tumor replication	OncoSG LUAD	KEAP1-or-NFE2L2 vs WT	7/162	1.94 (1.38 to 2.42)	8.18e-04	Statistical replication; non-TCGA LUAD support
External meta-analysis	CPTAC LUAD, CPTAC LSCC, OncoSG LUAD	KEAP1-or-NFE2L2 vs WT	3 cohorts	1.36 (1.14 to 1.58)	6.42e-34	Meta-analytic external tumor replication
Model-system support	DepMap/CCLE	KEAP1-or-NFE2L2 mutant vs WT NRF2 score	133/1,586	0.805	5.09e-18	Model-system state support; not clinical validation
Model-system support	DepMap/CCLE	NFE2L2 dependency in mutant vs WT	109/1,031	-0.301 gene effect	3.72e-07	Perturbational support; lower gene effect means stronger dependency

Fixed-effect meta-analysis of the three independent non-TCGA cohorts showed higher NRF2-state scores in KEAP1-mutant tumors (delta=1.24; 95% CI, 1.00 to 1.47; FDR = 1.11e-24) and in KEAP1-or-NFE2L2-altered tumors (delta=1.36; 95% CI, 1.14 to 1.58; FDR = 6.42e-34). The NFE2L2-specific estimate was supported primarily by CPTAC LSCC (delta=1.04; 95% CI, 0.56 to 1.53; FDR = 2.31e-05). These results establish a consistent external tumor-level association across lung adenocarcinoma and lung squamous cell carcinoma cohorts.

DepMap/CCLE provided a complementary model-system layer. Among 1,719 models with expression and metadata, NRF2 scores were higher in KEAP1-mutant models than in wild-type models (n=91 versus 1,628; delta=0.895; FDR = 1.09e-14), in NFE2L2-mutant models (n=52 versus 1,667; delta=0.516; FDR = 4.95e-05), and in combined KEAP1-or-NFE2L2-mutant models (n=133 versus 1,586; delta=0.805; FDR = 5.09e-18). Together, the external tumor cohorts and model-system analyses provide complementary evidence for the KEAP1/NFE2L2-to-NRF2 state axis.

### Benchmarking VISTA against simpler public-data workflows

A benchmark framework was prespecified to compare VISTA with mutation-only, ssGSEA-only, mutation plus ssGSEA, and mutation plus DepMap-query workflows. A mutation-only analysis defines altered groups without showing whether an alteration induces a coherent tissue state. An ssGSEA-only analysis scores NRF2 and immune programs without anchoring the result to a variant hypothesis. Mutation plus ssGSEA recovers the central genotype-to-RNA association; it still leaves open whether the signal has tissue-compartment localization, histopathologic correlates, cell-line dependency support, or pharmacogenomic links. A mutation plus DepMap query can nominate dependencies, although the model-system signal may be detached from tumor RNA context. VISTA’s added value is the organized mechanism chain and the separation of discovery, robustness, context, perturbation support, and reproducibility layers (**Table 3**).

**Table 3:** VISTA benchmark table.

Workflow	Variant anchoring	RNA-state scoring	Tissue/morphology context	Dependency or drug-response layer	Robustness and reproducibility	Benchmark interpretation
Mutation-only	Yes; KEAP1/NFE2L2 alteration groups	No	No	No	No	Alteration prevalence and group definition; no genotype-to-state mechanism chain
ssGSEA-only	No	Yes; NRF2 and immune/TME programs	No	No	No	State description without variant anchoring
Mutation + ssGSEA	Yes	Yes; direct mutation-to-NRF2 contrast	No	No	No	Recovers the central genotype-to-RNA association; cross-layer context remains unassessed
Mutation + DepMap query	Yes	No tumor RNA requirement	No	Model-system dependency nomination	No	Links variants to cell-line dependencies but separates model-system evidence from tumor tissue state
VISTA multimodal workflow	Yes; protein-region/AlphaMissense-informed prioritization plus case-level mutation indicators	Yes; frozen 16-gene NRF2 signature and immune/TME states	Yes; spatial/single-cell context and WSI morphology baseline	Yes; DepMap/Chronos dependency and PRISM/GDSC/CTRP response axes	Yes; project-adjusted models, bootstrap, permutation, leave-project sensitivity, external cohorts, manifests and derived tables	Auditable evidence chain from variant to tissue state, morphology, model-system support and pharmacogenomic hypotheses; evidence tiers remain explicit

### NRF2 stress is embedded in hypoxic, stromal, myeloid, and immune tissue-state contexts

After the central mutation-to-state association was established, VISTA asked whether NRF2 stress appeared within a broader tumor ecosystem context (**Figure 4**) (7, 9). RNA-state scoring separated immune/TME activation from hypoxia and NRF2 stress axes, making it unlikely that NRF2 activation was merely a proxy for global immune infiltration (8, 9, 15). External spatial analyses supported a tissue-context model in which NRF2 stress co-structured with hypoxia, while CAF/TGF-beta barrier programs colocalized with hypoxic tissue regions (25–27, 50). These spatial results are not genotype-matched to all TCGA mutation-to-state cases, yet they support a plausible NRF2/hypoxia/stromal co-niche (27, 45, 50).

**Figure 4:**
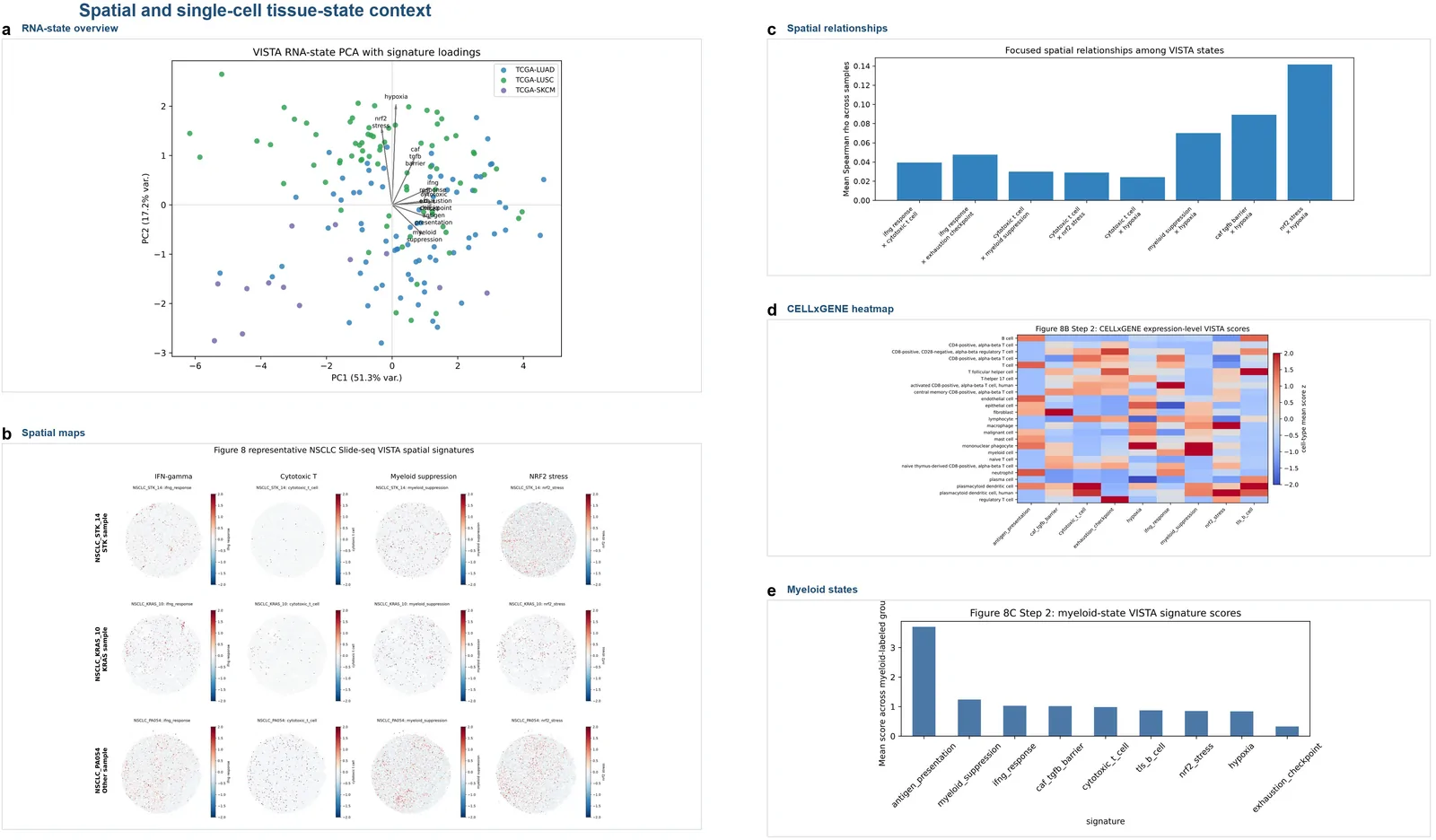
NRF2 stress tissue-state context from spatial and single-cell analyses. **(a)** RNA-state overview used to position NRF2 stress relative to hypoxia, stromal, myeloid and immune programs. **(b)** Representative spatial maps from GSE223501 showing the tissue distribution of VISTA signatures. **(c)** Focused spatial relationship summary for NRF2 stress and associated hypoxic, stromal and immune programs. **(d)** CELLxGENE expression-level cell-type heatmap for VISTA signature genes across annotated cell groups. **(e)** Myeloid and related expression-state summary supporting compartment-level interpretation. These analyses provide tissue-context support for NRF2-associated programs, although they are not genotype-matched causal validation.

Expression-level CELLxGENE scoring added compartmental resolution (22–24). Across two H5AD resources, VISTA scored 80,000 sampled cells. Cytotoxic and IFN-gamma programs localized to immune compartments (9, 17). Myeloid-suppression programs localized to myeloid, monocyte, and macrophage compartments (9, 17). CAF/TGF-beta programs localized to fibroblast and myofibroblast compartments (50, 51). Hypoxia/NRF2 stress signals appeared across malignant, stromal, and myeloid contexts (22, 39, 45). These patterns are consistent with a tissue-state model in which NRF2 stress interacts with hypoxic and stromal biology while coexisting with immune and myeloid programs (9, 45, 50).

### Immunotherapy cohorts define response-state relevance and transfer limits

VISTA then examined whether its RNA states carried response-relevant information in external immune-checkpoint blockade cohorts (**Figure 5**) (1, 28, 29). In GSE136961 NSCLC anti-PD-1 samples, a leave-one-out signature classifier predicted durable benefit with AUC 0.704 and bootstrap 95% CI 0.449 to 0.913, supporting response-state relevance in an external NSCLC setting (29). Static pretreatment transfer to GSE91061 melanoma was weak, with objective-response AUC 0.487 and disease-control AUC 0.472 (1). This negative transfer result is informative because it limits the claim: VISTA signatures should not be presented as a universal static pan-cancer pretreatment classifier (28, 29).

**Figure 5:**
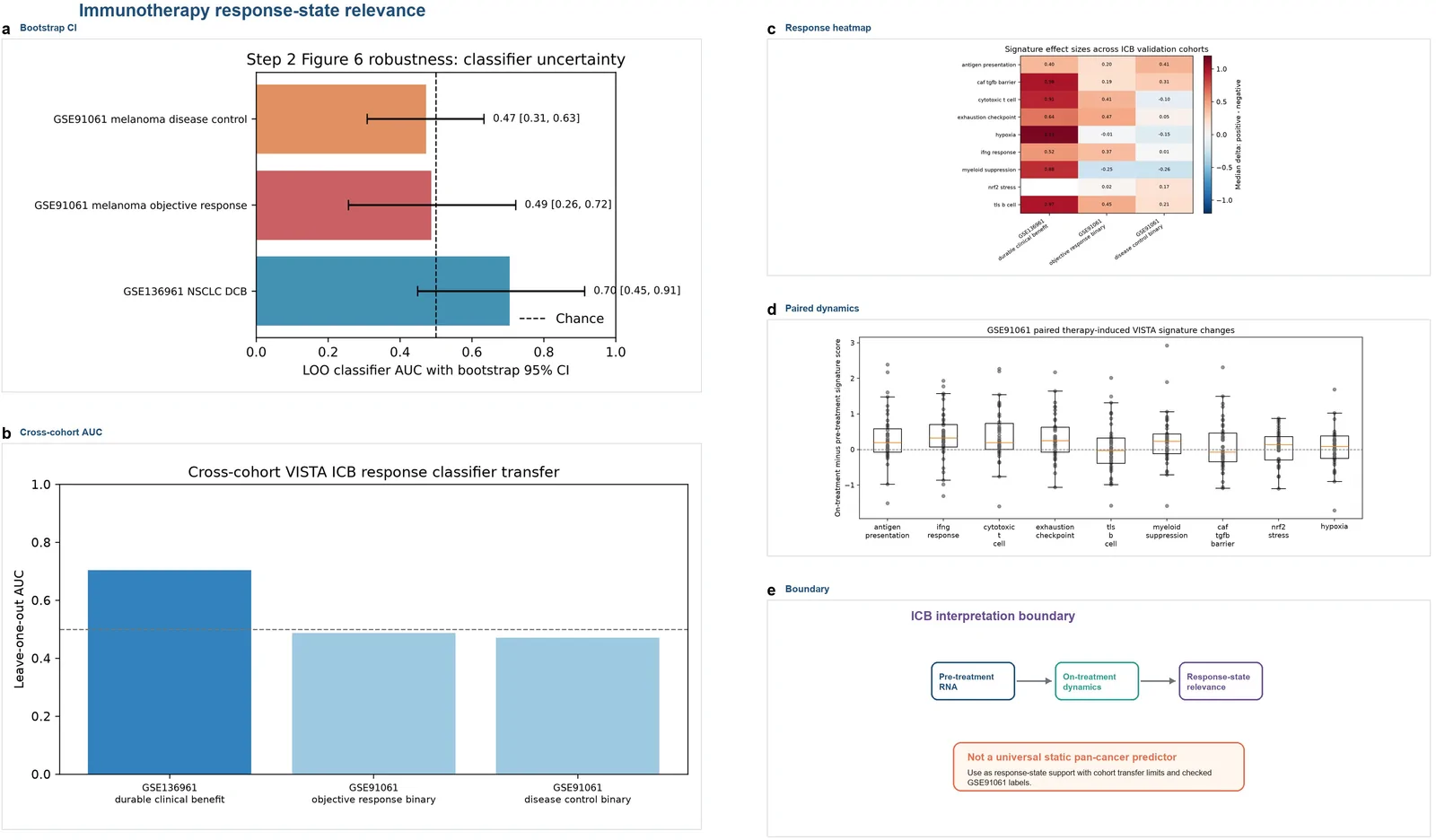
Immunotherapy response-state relevance and transfer limits. **(a)** Bootstrap confidence intervals for response-state classifier performance in the GSE136961 non-small-cell lung cancer immunotherapy cohort. **(b)** Cross-cohort area-under-the-curve summary illustrating static transfer performance and its limits. **(c)** Signature-response effect heatmap across cohorts and endpoints. **(d)** Paired on-treatment changes in GSE91061 melanoma samples using response labels finalized against Riaz et al. RECIST v1.1 definitions. **(e)** Interpretation boundary for immunotherapy analyses. The panels support response-state relevance of VISTA signatures without establishing a universal static pan-cancer immune-checkpoint predictor.

Paired melanoma biopsies in GSE91061 showed therapy-induced increases in immune-activation and checkpoint/exhaustion programs (1). Thus, VISTA signatures are better viewed as compact descriptors of immune state and treatment dynamics than as universal predictors (1, 28, 29). This boundary separates useful response-state biology from overbroad clinical prediction (28, 29).

### WSI feasibility analysis identifies exploratory tissue-state associations

For pathology integration, we analyzed a balanced feasibility set of 30 whole-slide images: 10 each from TCGA-LUAD, TCGA-LUSC, and TCGA-SKCM (**Figure 6**) (2–4). All SVS files were readable. Tissue-enriched tiles were encoded with Prov-GigaPath and aggregated using prespecified slide-level summary statistics (10) (**Table 4**). Exploratory associations between a pooled WSI component and RNA-defined tissue states included myeloid suppression (Spearman rho=0.584, FDR = 0.0357), hypoxia (rho=0.571, FDR = 0.0357), and the CAF/TGF-beta barrier program (rho=0.540, FDR = 0.0492). These findings establish a morphology-to-molecular feasibility baseline and nominate image features for evaluation in larger genotype-balanced cohorts. Future development will combine mutation-stratified sampling, independent site-level evaluation, pathology-guided regional annotation, and multimodal prediction of molecular tissue states.

**Figure 6:**
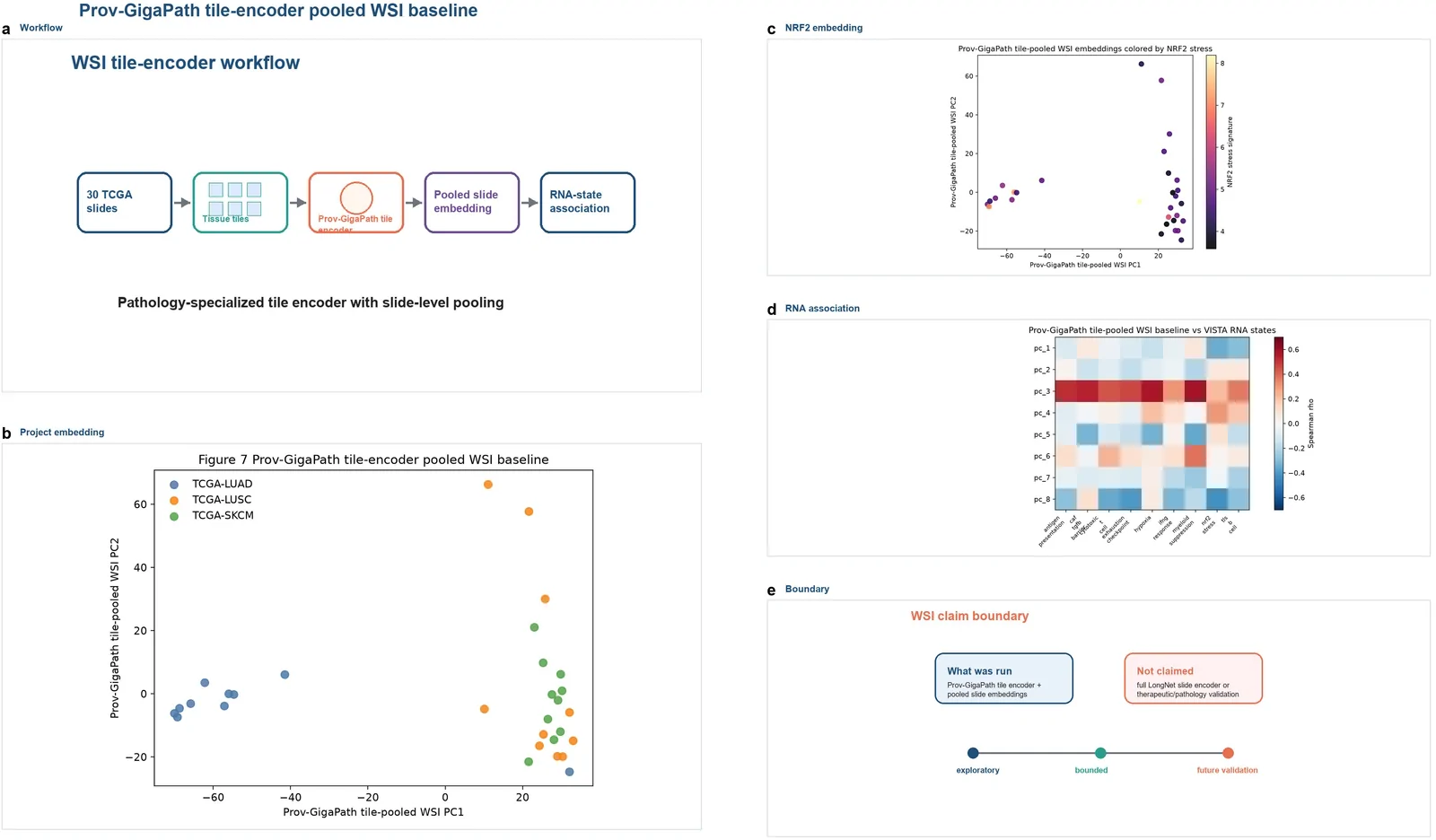
Prov-GigaPath tile-encoder pooled whole-slide image morphology baseline. **(a)** Balanced 30-slide whole-slide image pilot design and tissue-enriched tile-sampling workflow using the Prov-GigaPath pathology-specialized tile encoder. **(b)** Case-level pooled embedding scatter colored by TCGA project. **(c)** Embedding scatter colored by NRF2 stress score. **(d)** Association heatmap linking pooled tile-encoder components to RNA-state signatures in the 30-slide pilot. **(e)** Claim-boundary inset stating that this analysis demonstrates executable pathology-specialized tile-encoder morphology-to-molecular association. This is not full Prov-GigaPath LongNet slide-encoder inference and mutation-stratified morphology claims remain exploratory.

**Table 4:** Cross-resource drug-response axes.

Axis	Resources	Representative compounds	Interpretation boundary
HSP90/protein-homeostasis	PRISM, GDSC, CTRP	Tanespimycin, SNX-2112, luminespib	Cell-line sensitivity association
TXN/thioredoxin-redox	CTRP, PRISM	PX-12	Redox-axis nomination
ROS/piperlongumine-like	PRISM, GDSC, CTRP	Piperlongumine, PL-DI, curcumin	Stress-response association
SLC7A11/ferroptosis	PRISM, GDSC, CTRP	Erastin, buthionine-sulfoximine	Ferroptosis-related nomination
Proteasome/protein-degradation	PRISM, GDSC, CTRP	Bortezomib, MG-132, MLN2238	Protein-degradation association
DNA-damage/topoisomerase	PRISM, GDSC, CTRP	Doxorubicin, etoposide, camptothecin	Drug-response association only

### DepMap/Chronos and pharmacogenomic analyses nominate NRF2-circuit vulnerabilities

VISTA also integrated DepMap/CCLE release data, including model metadata, protein-coding gene expression, somatic mutations, CRISPR gene-effect scores, and CRISPR dependency calls, to test whether the tumor-derived NRF2 state axis appeared in model systems (**Figure 7**; **Table 4**) (12, 31–33). Across 1,719 models with matched metadata and expression profiles, all 16 NRF2 signature genes were available. NRF2-high and NRF2-low groups each contained 430 models. NRF2 scores were higher in KEAP1-mutant models than in wild-type models (n=91 versus 1,628; delta=0.895; FDR = 1.09e-14), higher in NFE2L2-mutant models (n=52 versus 1,667; delta=0.516; FDR = 4.95e-05), and higher in combined KEAP1-or-NFE2L2-mutant models (n=133 versus 1,586; delta=0.805; FDR = 5.09e-18). In the lung lineage subset, KEAP1-mutant models also had higher NRF2 scores, consistent with lineage-relevant model-system concordance.

**Figure 7:**
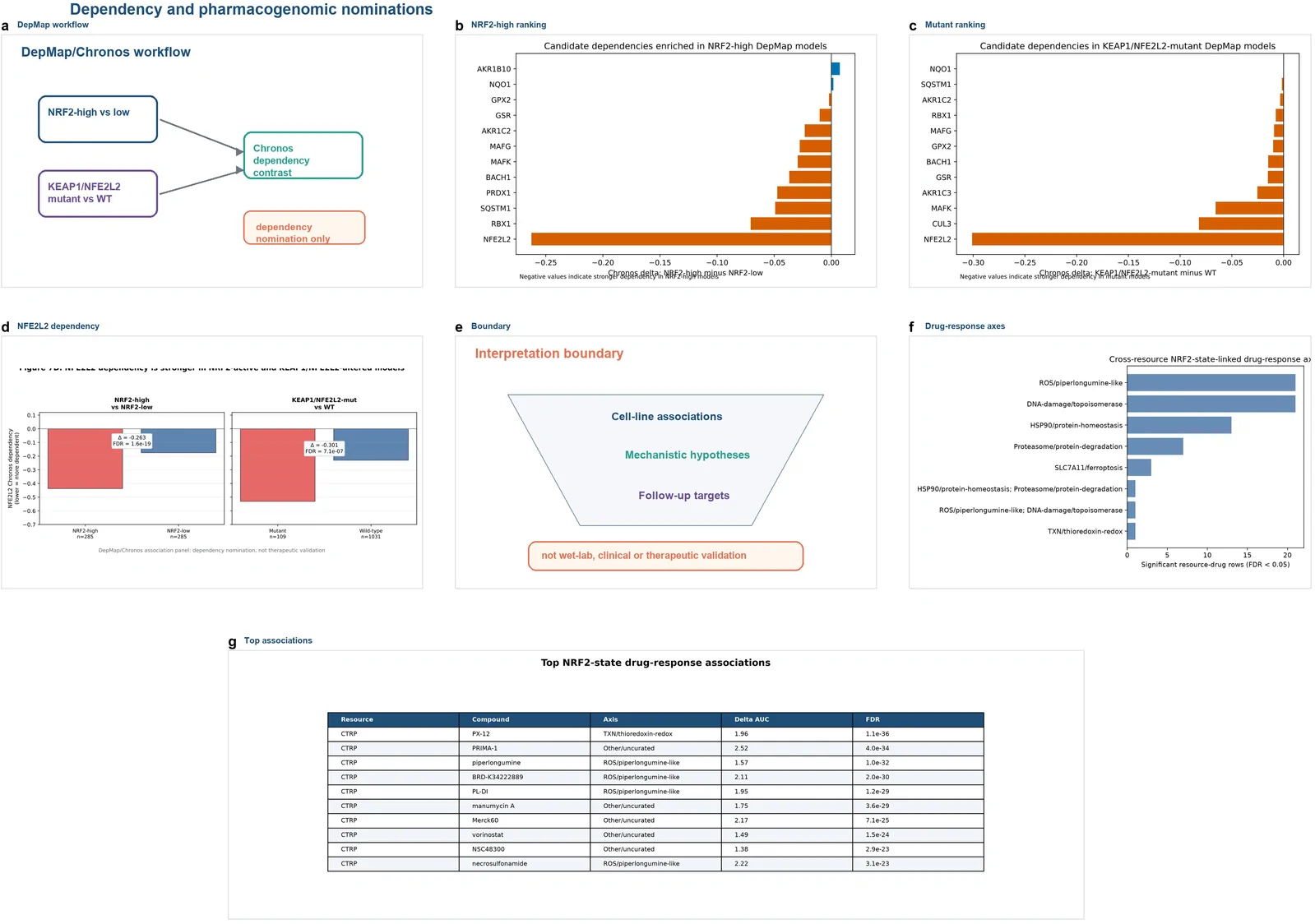
DepMap/Chronos and pharmacogenomic NRF2-circuit vulnerability nominations. **(a)** DepMap/Chronos workflow defining NRF2-high and NRF2-low expression groups, KEAP1/NFE2L2-mutant groups and gene-dependency contrasts. **(b)** Dependency ranking in NRF2-high cell lines. **(c)** Dependency ranking in KEAP1/NFE2L2-mutant cell lines. **(d)** Focused NFE2L2 dependency panel showing stronger dependency in NRF2-high and KEAP1/NFE2L2-mutant models. **(e)** NRF2-circuit interpretation schematic and claim boundary. **(f)** Cross-resource PRISM/GDSC/CTRP drug-response axis summary. **(g)** Representative top drug-response associations. These analyses nominate NRF2-circuit vulnerabilities and pharmacogenomic association axes from cell-line resources; they do not constitute therapeutic validation, clinical drug validation or wet-lab perturbation evidence.

Dependency testing pointed to an NRF2-circuit vulnerability pattern. NRF2-high models showed stronger NFE2L2 dependency than NRF2-low models (gene-effect delta=-0.255; FDR = 5.49e-19; Spearman rho=-0.312), and KEAP1-or-NFE2L2-mutant models showed stronger NFE2L2 dependency than wild-type models (delta=-0.301; FDR = 3.72e-07). Additional dependency contrasts implicated GCLM, GCLC, TXNRD1, SLC7A11, MAFK, and CUL3 in NRF2-high or KEAP1/NFE2L2-altered contexts. These results place NFE2L2 as both a state-defining transcriptional regulator and a model-system dependency in NRF2-activated cancer models (31, 39, 41). The DepMap/CCLE model-system validation is summarized in **Supplementary Figure S2**.

In VISTA, these findings extend the association from variant to RNA state and provide model-system and pharmacogenomic context; they do not establish therapeutic efficacy or clinical actionability.

## Discussion

VISTA recovers a coherent KEAP1/NFE2L2-to-NRF2 stress mechanism and connects it to tissue-state, morphology, clinical-response context, dependency, pharmacogenomic, and perturbation-support layers (10, 31, 39, 41). The strongest evidence remains the matched TCGA mutation-to-RNA association, which is stable under project adjustment, bootstrap confidence intervals, permutation testing, and leave-project sensitivity (2–4, 52). The KEAP1/NFE2L2-to-NRF2 axis is therefore the central mechanistic result, not a by-product of broad multimodal integration.

This study frames VISTA as a tissue-state modeling framework, not as a patient-specific simulator. It does not simulate individual patient trajectories or prospectively assign therapy. It integrates public molecular, tissue, histopathologic, dependency, and pharmacogenomic resources to reconstruct auditable links between variant biology, tissue state, and validation-ready hypotheses (5–7). This positioning matches the evidence generated by the present computational analysis.

TCGA provided the discovery setting, and CPTAC LUAD, CPTAC LSCC, and OncoSG LUAD independently replicated the association between KEAP1/NFE2L2 alterations and elevated NRF2 state scores (53, 54). Fixed-effect meta-analysis across these cohorts further supported the consistency of the external tumor-level association. LUAD-CAS and SMC lung contributed directionally concordant descriptive evidence from cohorts with fewer than three altered samples in the relevant contrasts. DepMap/CCLE extended this evidence into model systems by linking KEAP1/NFE2L2 mutation and an NRF2-high state to stronger NFE2L2 dependency. This tiered evidence structure connects discovery, independent tumor replication, descriptive cohort concordance, and model-system dependency within the same biological axis.

The benchmark analysis clarifies what VISTA adds beyond simpler workflows. A mutation-only workflow defines altered groups while missing tissue-state consequences. An ssGSEA-only workflow scores transcriptional states without variant anchoring (8, 15). Mutation plus ssGSEA captures the central association without creating a cross-layer evidence chain. Mutation plus DepMap query can nominate dependencies, although these may be disconnected from tumor context (12, 31). VISTA’s contribution is an auditable structure linking protein-informed variants, RNA states, tissue and morphology context, perturbation evidence, pharmacogenomic associations, and reproducibility materials (**Table 5**).

**Table 5:** WSI foundation-model benchmark design.

Benchmark component	Design specification	Primary output	Interpretation boundary
Pilot cohort	30 diagnostic TCGA whole-slide images; balanced design: 10 TCGA-LUAD, 10 TCGA-LUSC and 10 TCGA-SKCM	Case-level morphology-to-molecular feasibility benchmark	Exploratory morphology associations; not a clinical prediction cohort
Input and QC	SVS whole-slide files; locally readable slides selected from the 30-slide manifest	Record project, case ID, slide ID, dimensions, sampled tiles and runtime; retain input manifest	Raw WSI are provenance inputs and are not redistributed in the lightweight code package
Tissue sampling	Thumbnail-based tissue mask followed by tissue-enriched tile-origin sampling; maximum 8 tiles per slide in the pooled baseline	Reduce blank/background tiles while preserving a fixed, auditable sampling rule	Tile sampling is a computational baseline and does not constitute lesion-level annotation
Encoder	Pretrained Prov-GigaPath pathology-specialized tile encoder (`hf_hub:prov-gigapath/prov-gigapath`)	Encode 256-pixel crops after resize/center-crop to 224-pixel model input; raw tile embedding dimension 1,536	Use the term “pathology-specialized tile-encoder pooled baseline”; this is not full Prov-GigaPath LongNet slide-encoder inference
Slide aggregation	Per-slide mean, standard deviation, 25th percentile and 75th percentile across tile embeddings	6,144 pooled features per slide, followed by standardized PCA; report up to 8 principal components	Pooling summarizes slide-level morphology and does not provide spatial attention or cell-level localization
Molecular endpoints	Frozen VISTA RNA signatures: NRF2 stress, hypoxia, CAF/TGF-beta barrier, myeloid suppression, cytotoxic T-cell, IFN-gamma, exhaustion/checkpoint, antigen presentation and TLS/B-cell modules	Spearman correlation between WSI components and RNA-state scores; report sample size, rho, P value and Benjamini-Hochberg FDR	Associations are exploratory morphology-to-molecular links and do not establish causality
Genotype endpoints	KEAP1, NFE2L2, TP53, KRAS, STK11 and EGFR case-level mutation indicators	Compare embedding components between mutated and wild-type groups using Welch t tests with FDR correction where group sizes permit	Mutation-stratified morphology findings are exploratory and require larger independent validation
Reproducibility outputs	Case embedding matrix, tile manifest, molecular merged table, association tables, run metadata and figure source files	Record model name, pretrained status, tile size, tiles per slide, aggregation rule, device and runtime	Outputs support auditability and external re-analysis; they do not convert the feasibility benchmark into a clinical classifier

The 30-slide WSI analysis establishes a morphology-to-molecular feasibility baseline by linking Prov-GigaPath representations to myeloid suppression, hypoxia, and CAF/TGF-beta tissue states (10). The next stage of VISTA will evaluate these candidate imaging features in larger genotype-balanced cohorts with matched mutation, RNA-expression, clinical, and histopathologic data, using stratified sampling by cancer type and KEAP1/NFE2L2 alteration status, patient-level separation of training, model-selection, and evaluation sets, and independent institutions as site-held-out cohorts. Pathologist-guided annotation of tumor, stroma, necrosis, immune aggregates, and tissue artifacts, together with attention-based or multiple-instance learning, can enhance regional interpretability and identify tissue regions contributing most strongly to molecular-state estimates (18–21). Performance assessment will incorporate discrimination, calibration, confidence intervals, and subgroup consistency across histologic and technical strata. Scaling this component will require coordinated management of biological heterogeneity, technical variation, annotation quality, and computational demand through genotype-enriched case assembly, balanced tumor-type representation, standardized quality control, color normalization, stain augmentation, site-aware evaluation, regional sampling and aggregation that preserve spatial information, harmonized molecular labels, specimen-level matching, scalable image storage, tile extraction, foundation-model inference, cached embeddings, reproducible preprocessing, and access-controlled multicenter workflows, providing a practical route toward a reproducible image-based tissue-state biomarker within VISTA.

Several limitations remain. External tumor validation used public cBioPortal-accessible cohorts; other resources requiring authorization were not used as validation cohorts. LUAD-CAS and SMC lung cohorts were directionally supportive but underpowered for formal replication. Cell-line dependency and drug-response resources cannot reproduce tumor microenvironment biology, and spatial and single-cell resources provide compartmental context without genotype matching to the TCGA cases (22–27). The WSI analysis was limited by its small feasibility set. These boundaries are addressed through frozen preprocessing, prespecified validation criteria, evidence grading, and explicit separation between discovery, independent tumor replication, model-system support, and clinical inference.

## Methods

### Data acquisition and cohort definition

TCGA LUAD, LUSC, and SKCM data were assembled from public resources, including somatic mutation calls, RNA-seq-derived expression matrices, clinical metadata, and whole-slide image manifests (2–4). External immune-checkpoint blockade, spatial, single-cell, cBioPortal, and DepMap/CCLE resources were included as independent or supportive analysis layers according to data type and claim boundary (1, 12, 22, 25, 49, 53, 54).

### External validation cohorts

External tumor validation used cBioPortal-accessible cohorts with matched sample-level mutation and RNA expression data (55). Completed validation cohorts included CPTAC LUAD, CPTAC LSCC, OncoSG LUAD, LUAD-CAS, and SMC lung cancer cohorts; TCGA LUAD and LUSC cBioPortal studies were retained only as reference-overlap checks and not counted as independent validation. KEAP1 and NFE2L2 mutation status was assigned from mutation records, and NRF2 scores were computed from the frozen 16-gene NRF2 signature when genes were available in the expression profile. Within each cohort, gene-level expression values were z-scored across samples and averaged to obtain an NRF2-state score. Primary contrasts were KEAP1-mutant versus wild type, NFE2L2-mutant versus wild type, and KEAP1-or-NFE2L2-mutant versus wild type. Each contrast was summarized by mutant and wild-type sample counts, mean difference, bootstrap 95% confidence interval, Welch P value, FDR within contrast, direction concordance, and a replication flag. Cohorts with fewer than three mutant samples were labeled underpowered and interpreted as directional support only.

Model-system validation used DepMap/CCLE release data: Model.csv, OmicsExpressionTPMLogp1HumanProteinCodingGenes.csv, OmicsSomaticMutations.csv, CRISPRGeneEffect.csv, and CRISPRGeneDependency.csv (12, 30, 31, 34). NRF2 scores were computed using the same 16-gene NRF2 signature after expression z-scoring across models. NRF2-high and NRF2-low groups were defined by the upper and lower quartiles of NRF2 score. KEAP1 and NFE2L2 mutation indicators were derived from OmicsSomaticMutations.csv. Dependency analyses used CRISPR gene-effect scores, where lower values indicate stronger dependency. Primary model-system tests compared NRF2 scores between mutant and wild-type models and compared NFE2L2 and NRF2-circuit gene-effect scores between NRF2-high and NRF2-low groups and between KEAP1-or-NFE2L2-mutant and wild-type models. These analyses were interpreted as mechanistic or perturbational support rather than clinical tumor validation.

### Variant processing and protein-prior annotation

Somatic variants were harmonized to case-level mutation indicators for core cancer genes (56). Priority variants were mapped to protein regions, domain annotations, and AlphaMissense-informed priors where available (13, 14, 48). KEAP1/NFE2L2 were focused as the central mechanism axis based on regulatory coherence with NRF2 biology (39–41).

### RNA signature scoring

VISTA immune, TME, and stress signatures were scored from processed expression data (8, 15). Deconvolution and cancer immune-state references provided supporting context for immune/TME scoring (9, 16, 17). Signatures included NRF2 stress, hypoxia, CAF/TGF-beta barrier, myeloid suppression, cytotoxic T cell, IFN-gamma response, exhaustion/checkpoint, antigen presentation, and TLS/B-cell modules (9, 15, 17, 50). Case-level signature scores were merged with mutation and clinical metadata for downstream association testing (57–59).

### Robustness analysis

Mutation-to-signature associations were evaluated using group contrasts, project-adjusted models, age-adjusted sensitivity models where applicable, bootstrap confidence intervals, permutation testing, and leave-project sensitivity (52, 57). Multiple-testing correction and claim-strength labels were used to separate strong, supportive, and exploratory findings (52).

### Cross-layer evidence assessment and adjudication

Evidence was evaluated using prespecified categorical tiers based on five criteria: directness to the biological claim, sample and modality matching, cohort independence, statistical precision and robustness, and reproducibility of data processing. Matched tumor variant-to-RNA associations and independent tumor replication formed the primary evidence tier. Spatial and single-cell localization, DepMap/Chronos dependency, and external response-state analyses provided supportive evidence within their respective biological settings, whereas WSI and pharmacogenomic associations were classified as exploratory evidence. Each layer retained its modality-specific effect estimate, uncertainty measure, quality-control criteria, and claim boundary. Cross-layer concordance strengthened biological coherence. When findings differed across layers, the central interpretation followed the highest-tier evidence most directly aligned with the claim, while the differing result was retained as context-specific evidence and used to refine the scope of interpretation and priorities for subsequent validation. This categorical framework preserves the distinct biological questions addressed by tumor genomics, tissue morphology, model-system dependency, and pharmacogenomics.

### Immunotherapy cohort analysis

External ICB datasets were processed to evaluate response-state relevance (1, 28, 29). Classifier performance was summarized using leave-one-out or cohort-appropriate evaluation and bootstrap confidence intervals (52, 57). Static transfer limitations and paired treatment dynamics were reported separately (1, 29).

### Spatial and single-cell analyses

H5AD single-cell resources were scored using VISTA signatures (22–24). Spatial transcriptomics resources were scored with the same signature framework (25–27). Direct h5py access was used where possible for lightweight H5AD reading. These analyses were interpreted as tissue-context support, not genotype-matched causal validation (22, 25, 27).

### DepMap/Chronos dependency and drug-response association analysis

DepMap/CCLE model-system validation integrated Model.csv, OmicsExpressionTPMLogp1HumanProteinCodingGenes.csv, OmicsSomaticMutations.csv, CRISPRGeneEffect.csv, and CRISPRGeneDependency.csv (11, 12, 31, 33). NRF2-high and NRF2-low groups were defined from the NRF2 signature score using upper- and lower-quartile cutoffs (8, 15). Dependency differences were tested between NRF2-high and NRF2-low groups and between KEAP1/NFE2L2-mutant and wild-type groups using CRISPR gene-effect scores, where lower values indicate stronger dependency (31, 39, 41). Results were interpreted as model-system dependency support and vulnerability nomination rather than clinical validation (11, 12).

For orthogonal pharmacogenomic context, VISTA linked the same NRF2 cell-line state annotations to PRISM secondary-screen and GDSC1/GDSC2 fitted dose-response AUCs after cell-line name harmonization (30, 34–36). VISTA also linked NRF2 state annotations to CTRP v2 concentration-response curve AUCs (37, 38). Compound-level high-versus-low NRF2 contrasts, Spearman associations with continuous NRF2 score, and false-discovery-rate corrections were computed within each resource (52, 57). Cross-resource summaries grouped compounds by curated stress-response axes (30, 34, 37, 38). These analyses were interpreted as cell-line drug-response associations, not clinical therapeutic validation (35, 36, 38).

### Reproducibility

Analysis scripts, derived result tables, quality-control reports, figure source data, and data-acquisition manifests are provided with the study materials. Raw WSI, H5AD, DepMap/CCLE, and large expression files are not redistributed because of size and source-specific access constraints. Where applicable, data manifests record file sizes and SHA256 hashes.

## Data Availability

The original contributions presented in the study are included in the article/**Supplementary Material**. Further inquiries can be directed to the corresponding authors.

## Supplementary material

Supplementary Figure 1 External tumor-level validation of the KEAP1/NFE2L2-to-NRF2 state axis. Forest-style dot plot showing NRF2-state score differences between altered and wild-type tumors across independent cBioPortal-accessible validation cohorts and external fixed-effect meta-analysis. Points show altered-minus-wild-type differences; horizontal bars show bootstrap 95% confidence intervals for cohort-level analyses and fixed-effect 95% confidence intervals for meta-analysis. CPTAC LUA(D) CPTAC LSC(C) and OncoSG LUAD support higher NRF2-state scores in KEAP1- or KEAP1/NFE2L2-altered tumors. TCGA-overlap cohorts are not included in this external-validation plot.

Supplementary Figure 2 DepMap/CCLE model-system validation of NRF2-state and NFE2L2 dependency. **(A)** KEAP1-mutant, NFE2L2-mutant, and combined KEAP1-or-NFE2L2-mutant DepMap/CCLE models show higher NRF2-state scores than wild-type models. **(B)** NFE2L2 gene-effect dependency is stronger in NRF2-high models and in KEAP1/NFE2L2-mutant models. Lower gene-effect scores indicate stronger dependency. DepMap/CCLE analyses provide model-system support and are not interpreted as clinical tumor validation.
